# Genome assembly and transcriptome resource for river buffalo, *Bubalus bubalis* (2*n* = 50)

**DOI:** 10.1093/gigascience/gix088

**Published:** 2017-09-01

**Authors:** John L Williams, Daniela Iamartino, Kim D Pruitt, Tad Sonstegard, Timothy P L Smith, Wai Yee Low, Tommaso Biagini, Lorenzo Bomba, Stefano Capomaccio, Bianca Castiglioni, Angelo Coletta, Federica Corrado, Fabrizio Ferré, Leopoldo Iannuzzi, Cynthia Lawley, Nicolò Macciotta, Matthew McClure, Giordano Mancini, Donato Matassino, Raffaele Mazza, Marco Milanesi, Bianca Moioli, Nicola Morandi, Luigi Ramunno, Vincenzo Peretti, Fabio Pilla, Paola Ramelli, Steven Schroeder, Francesco Strozzi, Francoise Thibaud-Nissen, Luigi Zicarelli, Paolo Ajmone-Marsan, Alessio Valentini, Giovanni Chillemi, Aleksey Zimin

**Affiliations:** 1 The Davies Research Centre, School of Animal and Veterinary Sciences, University of Adelaide, Roseworthy, SA 5371, Australia; 2 AIA-LGS, Associazione Italiana Allevatori, Laboratorio Genetica e Servizi, Via Bergamo 292, 26100 Cremona (CR), Italy; 3 National Center for Biotechnology Information, National Library of Medicine, National Institutes of Health, Bethesda, MD 20894, USA; 4 Recombinetics, 1246 University Ave W, St Paul, MN 55104, USA; 5 USDA-ARS U.S. Meat Animal Research Center, 844 Road 313, Clay Center, NE 68933, USA; 6 IRCCS Casa Sollievo della Sofferenza, Bioinformatics Unit, S. Giovanni Rotondo, Italy; 7 Università Cattolica del Sacro Cuore, Via Emilia Parmense 84, 29122 Piacenza PC, Italy; 8 CNR, Istituto di Biologia e Biotecnologia Agraria Via Einstein, 26900 Lodi, Italy; 9 ANASB Associazione Nazionale Allevatori Specie Bufalina, Centuran, Caserta, Italy; 10 IZSM, Istituto Zooprofilattico Sperimentale del Mezzogiorno, Via Salute, 2–80055, Portici (NA), Italy; 11 Department of Pharmacy and Biotechnology (FaBiT), University of Bologna Alma Mater, Via Belmeloro 8/2, 40126 Bologna, Italy; 12 CNR, Istituto Per Il Sistema Produzione Animale In Ambiente Mediterraneo, Via Argine, 1085, 80147 Napoli, Italy; 13 Illumina, Inc. 499 Illinois St. Suite 210, San Francisco, CA 94158, USA; 14 Università degli Studi di Sassari, Piazza Università 21, 07100 Sassari, Italy; 15 USDA, ARS, Animal Genomics and Improvement Laboratory, Building 306 BARC-East, Beltsville, MD 20705-2350, USA; 16 Scuola Normale Superiore, Piazza dei Cavalieri 7, 56125 Pisa, Italy; 17 ConSDABI, Consorzio per la Sperimentazione, Divulgazione e Applicazione di Biotecniche Innovative, Contrada Piano Cappelle, Benevento (BN), Italy; 18 CRA Centro di Ricerca per la Produzione delle Carni ed il Miglioramento Genetico, Via Salaria 31, 00015, Montorotondo, Italy; 19 Merial Italia, via Lorenzini 8, 20139 Milano, Italy; 20 Dipartimento di Agraria, Università degli Studi di Napoli “Federico II”, via Università 100, 80055 Portici (NA), Italy; 21 Department of Veterinary Medicine and Animal Production, University of Naples Federico II, via Delpino 1, 80137 Napoli, Italy; 22 Department of Agriculture, Environment and Food, University of Molise; 23 Parco Tecnologico Padano, Via Einstein, 26500, Lodi, Italy; 24 Enterome, 94-96 Avenue Ledru-Rollin, 75011 Paris, France; 25 Universit à della Tuscia, Via S. Camillo de Lellis, 01100 Viterbo, Italy; 26 SCAI Super Computing Applications and Innovation Department, Cineca, Via dei Tizii 6, 00185, Rome; 27 University of Maryland, College Park, MD 20742, USA; 28 Present address: Human Genetics, Wellcome Trust Sanger Institute, Genome Campus, Hinxton, CB10 1HH, UK; 29 Present address: Irish Cattle Breeding Federation, Highfield House, Shinagh, Bandon, Co., Cork, P72 × 050, Ireland

**Keywords:** Water buffalo, genome assembly, transcriptome, annotation

## Abstract

Water buffalo is a globally important species for agriculture and local economies. A *de novo* assembled, well-annotated reference sequence for the water buffalo is an important prerequisite for studying the biology of this species, and is necessary to manage genetic diversity and to use modern breeding and genomic selection techniques. However, no such genome assembly has been previously reported. There are 2 species of domestic water buffalo, the river (2*n* = 50) and the swamp (2*n* = 48) buffalo. Here we describe a draft quality reference sequence for the river buffalo created from Illumina GA and Roche 454 short read sequences using the MaSuRCA assembler. The assembled sequence is 2.83 Gb, consisting of 366 983 scaffolds with a scaffold N50 of 1.41 Mb and contig N50 of 21 398 bp. Annotation of the genome was supported by transcriptome data from 30 tissues and identified 21 711 predicted protein coding genes. Searches for complete mammalian BUSCO gene groups found 98.6% of curated single copy orthologs present among predicted genes, which suggests a high level of completeness of the genome. The annotated sequence is available from NCBI at accession GCA_000471725.1.

## Data Description

### Background information on *Bubalus bubalis*

A reference genome sequence is important for understanding the biology of a species, managing genetic diversity, and, in the case of domestic animals, applying new genome-based selection methods for genetic improvement [1]. The water buffalo (*Bubalus bubalis*) was domesticated about 5000 years ago, and since then it has been of economic importance as a dairy, meat, and draught animal in most regions of the world [2]. There are 2 subspecies of water buffalo, known as the river and swamp types, which differ quite considerably both in terms of genetics and phenotypes. The river buffalo has 50 chromosomes whereas the swamp buffalo has 48 chromosomes, resulting from the fusion of buffalo chromosomes 4p with 9 [3]. River buffaloes are found from western Asia to Europe and have been selected for milk production. Swamp buffaloes are more common in eastern Asia and are used for meat and milk, although they only produce less than 500 L per lactation, and as a draught animal, they have undergone little genetic improvement. There are about 182 million water buffaloes in the world (compared to 1.3 billion cattle), with about 174 million in Asia, 3.7 million in Africa, 3.3 million in South America, and smaller populations in Europe and Australia. Recently the wild African buffalo (*Syncerus caffer*) genome was published [4]. A water buffalo pseudo-sequence has been reported, but it was created by aligning short sequence reads to the cattle reference genome and hence has many errors and unresolved structural variants and repeats [5].

Here we describe a *de novo* assembly of the river buffalo genome and the annotation of the assembled sequence. RNA-Seq data were produced from 30 tissues, which were used in the annotation and have been deposited in the NCBI SRA database under the project PRJNA207334. These data provide an initial draft reference sequence to facilitate genomic studies of water buffalo, a species that has global economic relevance. The data were produced by a small consortium with minimal funding, demonstrating the opportunities now presented by the rapidly advancing next-generation sequencing technologies.

### Chosen animal and sequencing

An inbred, female Italian Mediterranean buffalo (Olimpia) from a half-brother and half-sister mating was chosen for sequencing to increase the homozygosity level across the genome and simplify the genome assembly. Blood samples were collected by a qualified veterinary surgeon during routine handling for disease surveillance at her home farm. Animal work was carried out in compliance with Italian laws on animal experimentation and ethics (DL No. 116, 27 January 1992).

The karyotype was verified as normal by high-resolution R-banding (Fig. S1). Genomic DNA was extracted from blood using a commercial kit (Genomic tip 100/G; Qiagen, Venlo, the Netherlands). Whole-genome shotgun sequence (WGS) and mate pair reads with a target fragment size of 20 kb were produced on a Roche 454 FLX titanium instrument using kits and protocols supplied by the manufacturer (Roche Biosciences, Nutley, NJ, USA) and short paired end and mate paired libraries produced and sequenced on an Illumina GAIIx instrument. The data are summarized in Table 1.

**Table 1:** Sequencing data used for the water buffalo genome assembly. The coverage is calculated using an estimated genome size of 2.8 Gbp.

Sequencing technology	Mean library fragment length, bp	Mean read length, bp	Number of reads	Coverage
454 FLX	800	353	16 737 372	×2.1
454 FLX mate pair	20 000	229	4 821 352	×0.4
Illumina GAIIx paired end	400	96	2 122 738 136	×73
Illumina GAIIx mate pair	4000–6000	75	335 354 888	×9.0

Raw genomic sequence data are available from the SRA (PRJNA207334).

Most of the coverage was from the Illumina sequencing data; however, the Illumina reads were considerably shorter than the 454 WGS reads. The longer 454 single-end reads provided information to resolve short repeats, which contributed to greater sequence contiguity. The Illumina protocol for building jumping libraries did not reliably produce mate pairs longer than 6 kb, whereas the 454 protocol produced mate pair libraries up to 20 kb. The latter provided additional connectivity, which was essential for building the scaffolds.

### Assembly

The success of every assembly project depends on the quality control of the input data. Due to an amplification step in library production, 454 mate pair libraries have redundancy, such that independent molecules originating from the same ligated genomic sequence may be represented many times. This redundancy is a problem as one of the main assumptions made by most assemblers is that the coverage is uniform. To filter redundant data, the 454 sequences were initially mapped to the genome of a closely related species, in this case *Bos taurus* version Btau_UMD3.1 [6], using the NUCmer aligner [7]. Pairs of mated reads that all start and end at approximately the same position were identified, and only 1 of the redundant pairs was retained. This reduced the number of 454 mate pair reads available for the assembly from 2 410 676 to 1 870 392.

The *de novo* assembly of the water buffalo genome was carried out using the MaSuRCA assembler version 1.9.2 (MaSuRCA, RRID:SCR_010691) [8]. The Illumina paired end reads were error corrected using QuORUM (QuORUM, RRID:SCR_011840) [9]. Then the MaSuRCA assembler was used to create super-reads via k-mers, with many of the Illumina reads extended to the same super-read. The 2 122 738 136 Illumina paired end reads were reduced to 104 345 186 super-reads using a k-mer size of 57 to extend the paired end reads. The super-reads were 274 bp long on average and covered the genome at about ×10.

The Illumina mate-pair protocol relies on circularization of longer (3–6 kb) fragments with a biotinylated linker, followed by shearing. Fragments with biotin junctions are then recovered and sequenced using standard paired-end protocol producing pairs where 3΄ and 5΄ ends of the fragment are switched. When a sequence goes through the biotin junction, a chimeric read is produced. Such chimeric reads were filtered out by mapping them against the super-reads produced from paired end read data. Non-junction pairs may be formed when the biotin junction is close to one of the ends of a fragment. Such non-junction pairs were filtered out by aggressively trying to join the mate orientation with the super-reads. If a pair was joined with short separation, it was discarded. About 30% of initial mate pairs were retained after this filtering. The reads remaining after filtering were assembled with the Celera Assembler version 6.2, part of the standard MaSuRCA pipeline, followed by the MaSuRCA gap closer [8], which closed 23% of scaffold gaps.

The final quantitative genome assembly statistics are summarized in Table 2. This buffalo genome assembly is available at the *GigaScience* database and the NCBI [10].

**Table 2:** Assembly statistics of water buffalo.

Total sequence length	2 836 166 969
Total assembly gap length	74 388 041
Gaps between scaffolds	0
Number of scaffolds	366 983^a^
Scaffold N50	1 412 388
Scaffold L50	581
Number of contigs	630 368
Contig N50	21 938
Contig L50	35 881

^a^One of the scaffolds is the full mitochondrial genome.

### Transcriptome resources

Gene expression data were produced to aid the annotation process. Samples from 30 tissues were collected from 2 buffalo calves following humane euthanasia and stored in liquid nitrogen. RNA was prepared using a TRizol^TM^-based protocol (Thermofisher Scientific, Via G.B. Tiepolo, 18. I-20900 Monza MB, Italy) or RNeasy mini isolation kit (Qiagen, Venlo, the Netherlands), depending on the tissue. RNA integrity was assessed using an Agilent Technologies 2100 Bioanalyzer, and samples with an RNA Integrity Number (RIN) value greater than 8.5 were used to prepare libraries with an mRNA-Seq sample preparation kit (Illumina Inc. San Diego, CA, USA) according to the manufacturer's protocol. Sequencing of 2 × 100 paired-ends was carried out on an Illumina HiSeq 2000. An average of 53.6 million paired-end reads were produced per tissue. The quality of the data was explored by reconstructing transcript sequences to generate expressed contigs. The raw reads were processed and analyzed using a *de novo* transcriptome assembly pipeline based on the Trinity software [11], which gave 163K unique sequences across all tissues.

### Genome annotation

Annotation of genes, transcripts, and proteins was done using the NCBI Eukaryotic Genome Annotation Pipeline [12]. The NCBI pipeline operates on a RefSeq copy of the submitted assembly and produces RefSeq transcript and protein models, which are tracked with NCBI Gene IDs. Prior to annotation, the genome was first masked for repeats using WindowMasker [13]. Transcripts, RNA-Seq reads, and proteins that were publicly available in NCBI archival databases as of November 2013 were aligned with the masked assembly using splice-aware global alignment tools Splign [14] and ProSplign [15]. The evidence used as input for this annotation run included 3004 buffalo transcripts present in Genbank or EST records, 737 buffalo Genbank protein sequences, 35 660 human known RefSeq proteins (with NP_ prefix), 13 246 *Bos taurus* known RefSeq proteins, and 1.6 million RNA-Seq reads spanning 30 different tissues generated in this study. To address the volume of RNA-Seq data, only a single representative of 100% identical redundant raw short Illumina RNA-Seq reads from this study was aligned, and best-ranking alignments (95% identity and 90% coverage) with identical splice structure, consensus splice sites, and similar start and end points were collapsed into representative alignments. Rare introns, which may reflect background noise, were filtered out. Overlapping transcript, protein, and RNA-Seq alignments with compatible open-reading frames were assembled into putative models by Gnomon [16]. Partial putative models were then extended by Gnomon, using an HMM-based algorithm from GenScan (GenScan, RRID:SCR_012902) [17] to gain start or stop codons or fill small internal gaps. In some cases, Gnomon also introduced sequence insertions or deletions (InDels) in the models to retain the supported reading frame at genomic InDel locations. The frame-shift corrected models were annotated with a title prefix “PREDICTED: LOW QUALITY PROTEIN.” In addition, a small number of models was predicted *ab initio* by Gnomon. The final set of models selected from the Gnomon set excluded *ab initio* predictions with no strong hit to UniProtKB/SwissProt or to eukaryotic proteins in the nr database, predictions with high homology to transposable or retro-transposable elements and poorly supported predictions conflicting with better-supported models annotated on the opposite strand. In addition, for genes with multiple alternative variants, the model selected had to be supported in its entirety by a single long alignment (e.g., a full-length mRNA) or by RNA-Seq reads from a single sample.

Protein naming was based on human orthologs, or lacking that, on best protein similarity; human orthologs were identified based on shared best Swiss-Prot alignments for the human RefSeq protein and the buffalo protein model, as well as considerations of local conservation of synteny [18]. The gene “type” categorization (e.g., protein-coding, pseudogene, non-coding RNA locus) was determined based on observed open reading frame lengths, statistical coding propensity, protein alignments, and protein orthology to human orthologs.

The final RefSeq annotation contains a total of 21 711 protein-coding genes (Table 3) and is collectively called *Bubalus bubalis* Annotation Release 100, which is available in the NCBI Nucleotide, Protein, and Gene resources database [10]. Additional RefSeq annotation details are available online [19].

**Table 3:** Counts of predicted genomic features in Annotation Release 100

Feature	Count
Genes and pseudogenes	27 837
Protein-coding	21 711
Non-coding	2303
Pseudogenes	3823
mRNAs	41 486
Fully supported	38 378
With > 5% *ab initio*	1662
Partial	1500
Other RNAs	5544
Fully supported	3911
With > 5% *ab initio*	0
Partial	0
CDSs	41 665
Fully supported	38 378
With > 5% *ab initio*	1956
Partial	1515

### Sequence repeats

Repeats in the buffalo genome assembly were identified using the RepeatMasker suite (RepeatMasker, RRID:SCR_012954) [20]. Scanning the buffalo genome assembly for cattle repeats as defined in the Repbase database [21] identified more than 3.8 million repetitive elements (Table S1). The majority of these repeated elements belong to the short interspersed elements (SINE > 2 million) and long interspersed elements (LINE > 1.1 million) families. In total, more than 1.2 giga base pairs (Gbp) of the buffalo genome assembly belong to repetitive elements, corresponding to 43.69% of the whole assembly.

### Genome assembly quality and annotation assessment

The genome assembly is made up of 366 983 scaffolds, with N50 of about 1.4 Mbp. A plot on the cumulative length of scaffolds shows that the 3669 longest scaffolds, less than 1% of all scaffolds, accounts for approximately 92% of the assembly size (Fig. S2). Most of the sequence assembled into the buffalo scaffolds shows a good match with *Bos taurus* genome; however, around 15% does not find a match with the cow genome and is either a sequence missing from the cow assembly or a putative buffalo-specific genomic sequence.

In addition to measuring standard assembly metrics, genome assembly and annotation quality were further assessed in several ways. The assembled buffalo sequence was compared with other closely related domestic species including cattle, goat, sheep, and pig (Table 4). All genomes in the table were annotated by NCBI, and the average genome size is approximately 2.8 Gbp. The total number of annotated proteins in buffalo is most similar to cattle (21 295), followed by goat (20 755), sheep (20 645), and then pig, the latter of which has more annotated protein coding genes (24 205). The number of partial coding sequence (CDS) is an indication of the quality of the genome annotation; the smaller the number of partial CDS, the better the annotation. The recently published goat genome [17] was assembled using the latest sequencing technologies and hence has high annotation quality, with only 455 partial CDS. Surprisingly, the buffalo genome assembly described here has fewer partial CDS than cattle and pig (see Table 4), considering that it was assembled using a combination of data from older 454 and Illumina short reads. In addition, as indicated above, more protein coding genes were identified in the annotation of the buffalo genome than the annotations of the genomes of cow, sheep, and goat.

**Table 4:** Genome annotation comparison with other domestic species.

Species	Common	Protein	Partial	Assembly	Divergence time	RefSeq assembly	Annotation
	name	coding genes	CDS	size	to buffalo, Myr	accession	release ID
*Bubalus bubalis*	Water Buffalo	21 711	1515	2 836 166 969	-	GCF_000471725.1	100
*Bos Taurus*	Cattle	21 295	1589	2 670 139 648	12.3	GCF_000003055.6	105
*Capra hircus*	Goat	20 755	457	2 922 813 246	24.6	GCF_001704415.1	102
*Ovis aries*	Sheep	20 645	758	2 615 516 299	24.6	GCF_000298735.2	102
*Sus scrofa*	Pig	24 205	4112	2 808 525 991	62	GCF_000003025.5	105

The completeness of the genome assembly was assessed using the Benchmarking Universal Single-Copy Orthologs (BUSCO, RRID:SCR_015008) [22]. The presence of the 4104 mammalian BUSCO gene groups was tested against the predicted buffalo gene set. The longest protein isoforms for each of the predicted nuclear genes were used as input for BUSCO searches. In total, 4048 of the BUSCO groups were present in the assembly, with an additional 50 fragmented genes and only 6 completely missing BUSCO groups (Table 5). The presence of 98.6% complete mammalian BUSCO gene groups suggests a high level of completeness of this buffalo genome assembly.

**Table 5:** Completeness of buffalo genome assembly as assessed by BUSCO.

Complete BUSCOs (C)	4048
Complete and single-copy BUSCOs (S)	4007
Complete and duplicated BUSCOs (D)	41
Fragmented BUSCOs (F)	50
Missing BUSCOs (M)	6
Total BUSCO groups searched	4104

## Conclusion

This is the first *de novo* sequence assembly and annotation of the river buffalo genome, which represents an important resource for this species and is a significant improvement on the previous alignment of low-coverage short reads to the bovine genome sequence.

## Availability of supporting data

Genome annotation results are available at the NCBI (ftp://ftp.ncbi.nlm.nih.gov/genomes/Bubalus_bubalis/), and supporting materials, which include transcripts and genome assembly, are available at the *GigaScience* database, *Giga*DB [10].

## Additional files

Additional file 1: Table S1. RNA-Seq dataset used for annotation.

Additional file 2: Figure S1. Karyotype of the chosen animal.

Additional file 3: Figure S2. A plot of cumulative scaffold length.

## Abbreviations

CDS: Coding sequence; BUSCO: Benchmarking Universal Single-Copy Orthologs; Gbp: giga base pairs; LINE: long interspersed elements; Mbp: maga base pairs; Myr: million years; NCBI: National Center for Biotechnology Information; nr database: non-redundant database; RefSeq: Reference Sequence; RNA-Seq: high-throughput messenger RNA sequencing; SINE: short interspersed elements; SRA: Sequence Read Archive; WGS: whole genome sequencing

## Competing interests

All authors declare that they have no competing interests.

## Funding

Funding for DNA sequencing for the water buffalo genome assembly was from members of the Italian Buffalo Genome Consortium (Parco Tecnologico Padano; Università degli Studi del Molise; CNR, Istituto di Biologia e Biotecnologia Agraria; CNR, Istituto Per Il Sistema Produzione Animale In Ambiente Mediterraneo, project CISIA-VARIGEAV; Consorzio per la Sperimentazione, Divulgazione e Applicazione di Biotecniche Innovative; CRA Centro di Ricerca per la Produzione delle Carni ed il Miglioramento Genetico; Istituto Zooprofilattico Sperimentale del Mezzogiorno; Università della Tuscia; Università Cattolica del Sacro Cuore; Università degli Studi di Napoli Federico II; Università degli Studi di Sassari and Università degli Studi di Udine) and for TPLS by USDA-ARS Project Plan 5438–31000-073–00D. The RNAseq data production was funded by the GenHome project from the Italian Ministry of Education. The genome annotation work carried out by NCBI was supported by the Intramural Research Program of the NIH, National Library of Medicine.

## Author contributions

J.L.W. and D.I. conceived of and managed the project; A.C., L.R., and V.P. selected Olimpia for sequencing; N.M., F.C., P.R., and B.M. collected and processed samples from Olimpia; L.I. verified Olimpia's normal karyotype; T.S. and T.P.L.S. produced the sequencing data. MMc created Illumina jumping libraries; S.S. checked and managed Illumina sequence data. A.Z., G.C., F.F., T.B., and G.M. carried out the genome assembly; N.M., D.I., B.C., F.P., and J.L.W. collected samples for transcriptome analysis; D.I., P.R., and F.S. produced the RNA-Seq data. F.S., L.B., M.M., and R.M. verified the RNASeq data quality; K.P. and F.T.N. annotated the assembled genome; W.Y.L., N.M., S.C., and R.M. assessed the assembly and annotation quality; J.L.W., A.V., P.A.M., L.Z., and D.I. oversaw the project, and C.L. and D.M. assisted in coordination of activities; J.L.W. and W.Y.L. drafted the manuscript, and all authors read, edited, and approved the final manuscript.

## Supplementary Material

Additional files
