# Supplementary figures and images for: Genome assembly and transcriptome resource for river buffalo, Bubalus bubalis (2n = 50)
Source: Gigascience. 2017 Sep 1;6(10):1–6. doi: 10.1093/gigascience/gix088 (PMC5737279; doi:10.1093/gigascience/gix088)

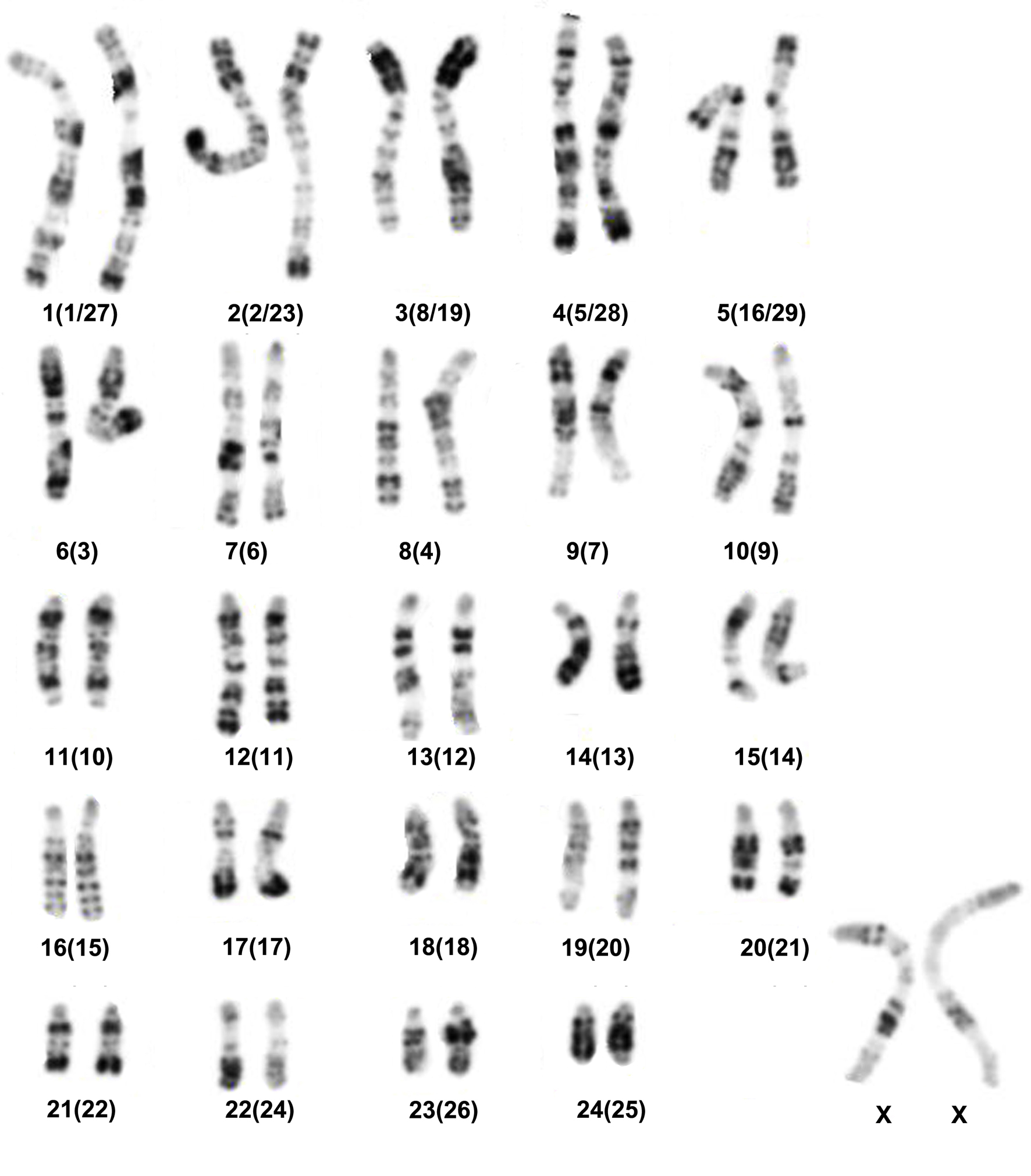

Supplement: Additional files [file gix088_supp.zip › Additional_File_3_FigS1_Olimpia_Karyotype_revised.jpg]

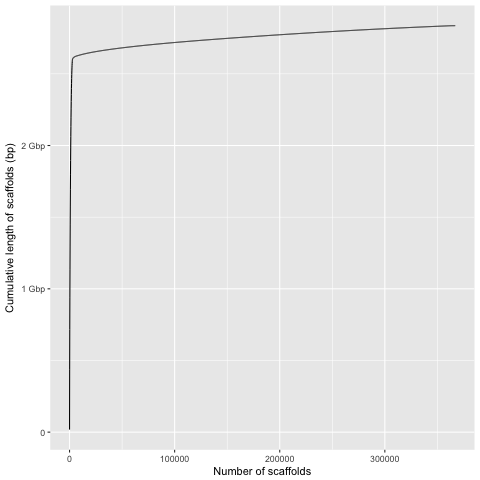

Supplement: Additional files [file gix088_supp.zip › Additional_File_4_FigS2_Cummulative_Scaffold_Length.tiff]
